# First-Line [^177^Lu]Lu-PSMA-617 Therapy Without ADT, Chemotherapy, or Local Therapy for Metastatic Hormone-Sensitive Prostate Cancer

**DOI:** 10.2967/jnumed.125.270704

**Published:** 2025-11

**Authors:** Vinay K. Giri, Gerald M. Kolodny, Marc B. Garnick

**Affiliations:** 1 Harvard Medical School, Boston, Massachusetts;; 2 Department of Medicine, Division of Medical Oncology, Beth Israel Deaconess Medical Center, Boston, Massachusetts;; 3 Department of Radiology, Division of Nuclear Medicine, Beth Israel Deaconess Medical Center, Boston, Massachusetts

**Keywords:** first-line [^177^Lu]Lu-PSMA-617, radiopharmaceutical, metastatic, prostate cancer

## Abstract

The treatment of metastatic prostate cancer relies on the use of androgen deprivation therapy, which is associated with many side effects, including adverse cardiovascular outcomes. ^177^Lu-vipivotide tetraxetan ([^177^Lu]Lu-PSMA-617) is used in the treatment of prostate-specific membrane antigen–expressing metastatic castration-resistant prostate adenocarcinoma. We present the case of a patient with prostate cancer with numerous metastatic lesions who declined androgen deprivation therapy, chemotherapy, and local therapy to the prostate gland and instead received first-line systemic [^177^Lu]Lu-PSMA-617. He exhibited a significant response in both the primary cancer and its metastatic foci after 2 doses of the treatment.

## Visual Abstract

**Figure:**
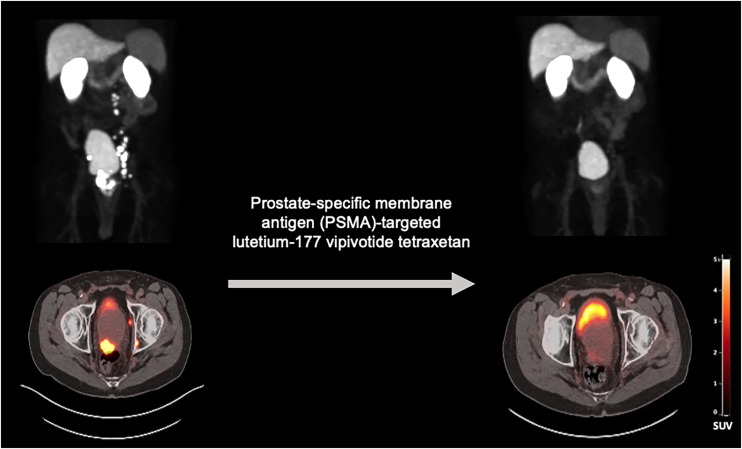

The up-front treatment of metastatic prostate adenocarcinoma primarily consists of androgen deprivation therapy (ADT) in combination with androgen receptor pathway inhibitors (ARPIs) with or without taxane-based chemotherapy. ^177^Lu-vipivotide tetraxetan ([^177^Lu]Lu-PSMA-617) is indicated for prostate-specific membrane antigen (PSMA)–expressing metastatic castration-resistant prostate cancer (mCRPC) after progression on ADT and ARPIs and can be administered before chemotherapy in patients for whom deferral of taxane-based therapy is appropriate (1).

[^177^Lu]Lu-PSMA-617 is a radiopharmaceutical β-emitter that targets the transmembrane glycoprotein PSMA. In the phase 3 VISION trial, [^177^Lu]Lu-PSMA-617 plus ADT improved overall survival compared with protocol-permitted standard of care, primarily an ARPI switch, in mCRPC after progression on an ARPI and taxane-based chemotherapy (2). In the phase 3 PSMAfore trial, [^177^Lu]Lu-PSMA-617 demonstrated significantly improved radiographic progression-free survival compared with an ARPI switch in patients who had not previously received chemotherapy for their metastatic disease (3). Other later-line studies of [^177^Lu]Lu-PSMA-617 include TheraP, in which [^177^Lu]Lu-PSMA-617 demonstrated an improved prostate-specific antigen (PSA) response versus cabazitaxel for mCRPC after progression on docetaxel (4), and ENZA-p, in which [^177^Lu]Lu-PSMA-617 in mCRPC improved overall survival in combination with enzalutamide versus an ARPI alone (5).

In the VISION trial, PSMA expression via whole-body tumor SUV_mean_ was the best predictor of treatment efficacy (6). Given the reduction of PSMA expression after the initiation of ADT, there is a biologic rationale for investigating [^177^Lu]Lu-PSMA-617 in earlier settings (7). In the phase 2 UpFront PSMA trial, patients with metastatic hormone-sensitive prostate cancer (mHSPC) who received ADT plus 2 cycles of [^177^Lu]Lu-PSMA-617 before docetaxel had improved rates of undetectable PSA at 48 mo compared with those who received ADT plus docetaxel (41% vs. 16%, respectively) (8).

The use of [^177^Lu]Lu-PSMA-617 before ADT for metastatic prostate cancer has been described in only a few published cases. One individual with metastatic recurrence of prostate cancer after radical prostatectomy and adjuvant radiation was treated with 2 cycles of [^177^Lu]Lu-PSMA-617 with complete radiographic response (9). In a pilot study by Privé et al., 10 patients with low-volume mHSPC were treated with 2 cycles of [^177^Lu]Lu-PSMA-617 without concurrent ADT. Half of the participants demonstrated a PSA reduction of greater than 50%, and 1 patient had a complete radiographic response (10).

This was followed by the phase 2 BULLSEYE trial, in which patients with oligometastatic prostate cancer with fewer than 5 metastatic sites and who previously received local therapy were randomized to receive up to 4 cycles of [^177^Lu]Lu-PSMA-617 or active surveillance with deferred treatment (11). Up-front treatment with [^177^Lu]Lu-PSMA-617 improved progression-free survival and was well tolerated without the development of any serious side effects that would preclude future systemic therapies (11).

Although these studies describe patients with oligometastatic prostate cancer, we present a case of up-front [^177^Lu]Lu-PSMA-617 administered to an individual with greater disease burden, including significant abdominopelvic metastatic lymphadenopathy and an osseous metastasis, who did not receive any initial local therapy to the prostate. The patient desired to avoid ADT because of its side-effect profile and his history of myocardial infarction. He also opted against primary therapy to the prostate gland to avoid possible complications from radiation or surgery, prompting the choice of first-line [^177^Lu]Lu-PSMA-617, and a response was seen in both the primary lesion and the metastases.

## CASE REPORT

Our patient was diagnosed with prostate adenocarcinoma at the age of 54 y. A screening PSA level in October 1996 was found to be 5.7 ng/mL. A 7-core transrectal prostate biopsy revealed Gleason score 3 + 3 adenocarcinoma in the right apex. A CT of the chest, abdomen, and pelvis and bone scintigraphy did not reveal metastatic disease. The patient opted for surveillance via PSA and MRI monitoring without additional biopsies. He did not receive surgery, radiation, or hormonal therapy.

**FIGURE 1.**
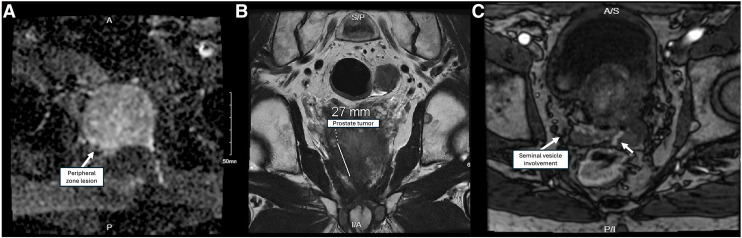
Progression of primary prostate tumor seen on MRI. (A) Subcentimeter lesion in right posteromedial peripheral zone seen in 2014 and 2016. Two other subcentimeter lesions not seen here are present in right posteromedial peripheral zone. (B) By 2024, this lesion grew to 2.7 cm and expanded from base to apex, crossing midline to left. (C) Bilateral lesions that involve right seminal vesicle and potentially left seminal vesicle.

His PSA slowly rose over the following years, with growth of a palpable prostate nodule and mildly worsening urinary obstructive symptoms. In 2008, he developed a non-ST-elevation myocardial infarction and underwent a 5-vessel coronary artery bypass graft surgery. By 2018, his urinary symptoms worsened, and finasteride was initiated for intermittent urinary retention (Fig. 1). By 2021, his PSA level increased to 87 ng/mL, and regional lymphadenopathy was noted on ^18^F-fluciclovine PET/CT. The patient declined radiation treatment to the prostate and lymph nodes.

By late 2023, his disease had spread to additional retroperitoneal and pelvic lymph node chains. A small acetabular lesion seen in December 2023 was markedly larger and more avid by June 2024, with an SUV_max_ of 12.7 on a ^18^F-piflufolastat PSMA PET/CT. A circulating tumor DNA sequencing panel revealed only low-level mutations in *JAK2* V617F (variant allele frequency, 0.26%) and *GNAS* R201C (variant allele frequency, 6.7%). Germline genetic testing did not reveal any pathogenic mutations.

The patient continued to decline ADT, ARPI therapy, chemotherapy, and local therapy to the prostate gland, and he eventually received off-label [^177^Lu]Lu-PSMA-617 monotherapy. After receiving his first dose of 7.46 GBq of [^177^Lu]Lu-PSMA-617, his urinary obstructive symptoms briefly worsened but then fully resolved. His pretreatment PSA level of 303.0 ng/mL declined to 33.6 ng/mL after the first dose and declined further, to 14.07 ng/mL, after a second dose (7.49 GBq) of [^177^Lu]Lu-PSMA-617.

**FIGURE 2.**
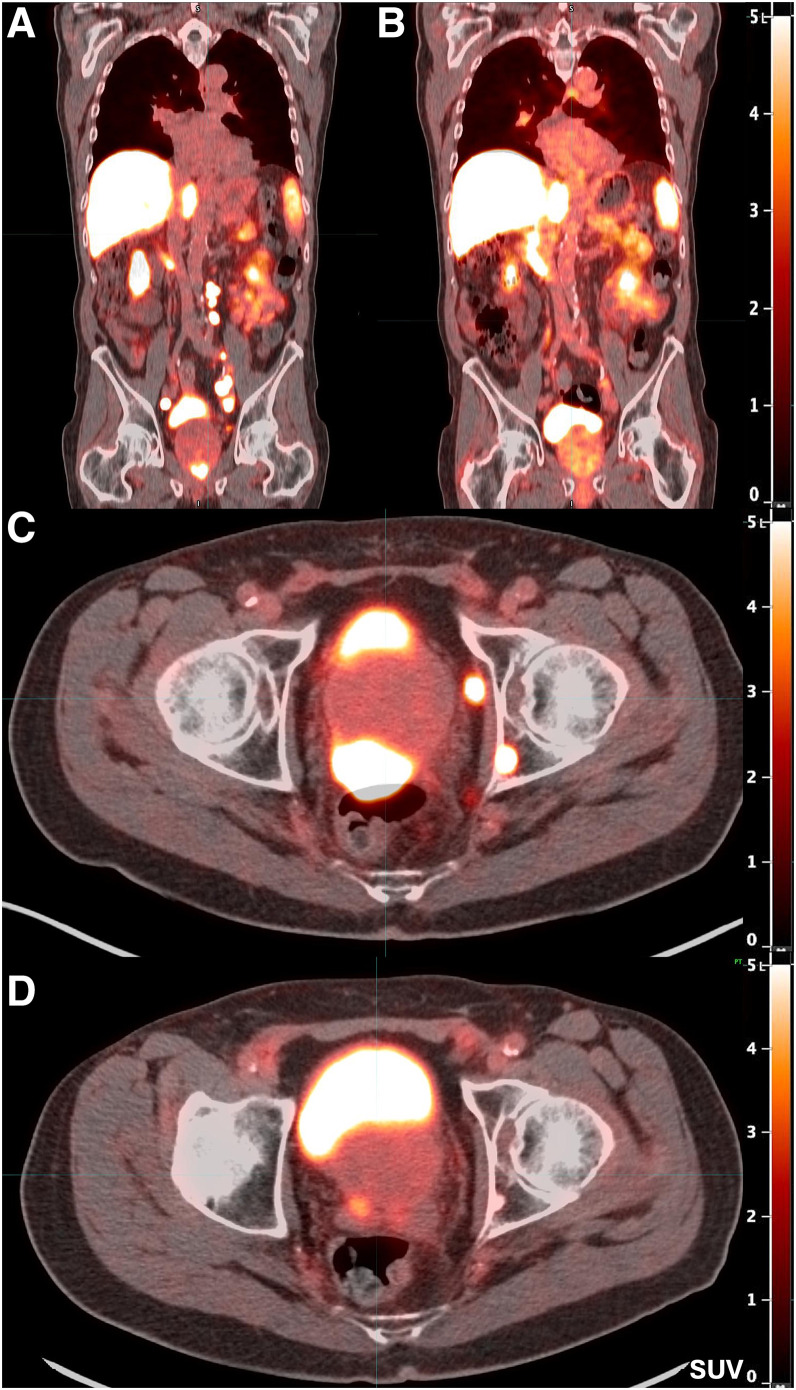
Burden of PSMA-expressing prostate cancer before and after treatment with 2 cycles of [^177^Lu]Lu-PSMA-617. (A and B) Reduction in overall disease burden is evident after treatment. Avidity of patient’s primary prostate tumor and his osseous metastasis shown before (C) and after (D) treatment.

A follow-up ^18^F-flotufolastat PSMA PET/CT scan performed after 2 doses of [^177^Lu]Lu-PSMA-617 demonstrated decreased avidity of the posterior left acetabular sclerotic metastasis (SUV_max_ decreased from 13 to 3.1), size and avidity of previously bulky retroperitoneal and bilateral pelvic lymphadenopathy (SUV_max_ decreased from 21–22 to 1.5–3.4), and radiotracer uptake in the posterior prostate (SUV_max_ decreased from 76 to 6). A repeat ^18^F-piflufolastat PSMA PET/CT 8 wk later demonstrated continued improvement in radiotracer avidity, including in the sclerotic acetabular lesions, which demonstrated background avidity, and only residual low-level uptake was noted in the prostate and pelvic sidewall lymph nodes (Fig. 2).

After 2 doses of [^177^Lu]Lu-PSMA-617, the patient declined any further treatment. He continues to have a prolonged PSA response, with a PSA level of 16.60 ng/mL measured 10 mo after the start of treatment, although this does not meet the threshold of 0.2 ng/mL, a favorable prognostic indicator historically used for individuals receiving ADT with or without an ARPI or chemotherapy. He developed mild thrombocytopenia, with a platelet count of 118,000 cells/μL (down from a baseline of 150,000–170,000 cells/μL) after his first dose, and mild anemia, with a hemoglobin level of 12.5 g/dL (down from a baseline of 14–15 g/dL) several months after his second dose. He has not experienced any additional side effects from [^177^Lu]Lu-PSMA-617, including renal toxicity, and he continues to have marked improvement in his obstructive urinary symptoms.

## DISCUSSION

Our patient with mHSPC and no prior local therapy to the prostate gland experienced a 95% reduction in PSA level and an improvement in urinary symptoms after 2 cycles of [^177^Lu]Lu-PSMA-617. His sites of disease included a pelvic osseous metastasis, multistation bulky lymphadenopathy, and a primary lesion in the peripheral zone of the prostate. This case differs from the oligometastatic disease evaluated in the BULLSEYE trial, as our patient did not have primary treatment to the prostate and had many more sites of metastatic involvement (11).

Although over 90% of hormone-naïve cases of prostate cancer uniformly express PSMA, over 15%–20% of patients with castration-resistant disease will develop PSMA-negative lesions (12). Therefore, by the time [^177^Lu]Lu-PSMA-617 is indicated in current standard of care settings, some patients are no longer eligible for this therapy. Although there may be a proven role in the future for the use of up-front [^177^Lu]Lu-PSMA-617 in individuals who prefer to avoid the potential complications or side effects associated with surgery, radiation, or ADT, caution is warranted, as long-term outcomes have not yet been compared in this setting.

To this end, trials are now evaluating the earlier use of [^177^Lu]Lu-PSMA-617. The phase 3 PSMA-DC trial will assess the efficacy of [^177^Lu]Lu-PSMA-617 in delaying disease progression after stereotactic body radiotherapy in patients with oligorecurrent prostate cancer (13). The phase 3 PSMAddition trial is assessing the efficacy of [^177^Lu]Lu-PSMA-617 added to up-front ADT plus an ARPI in mHSPC (14). Additionally, a PSMA-targeted radiopharmaceutical using the α-particle–emitting ^225^Ac demonstrated efficacy in patients with mHSPC who declined ADT, with 86% of patients experiencing a decline of more than 50% in their PSA level (15).

An approach of first-line radiopharmaceutical therapy with consolidative radiation to the prostate, if needed, might enable patients with a history of cardiac disease to avoid the cardiovascular complications associated with ADT. However, it is unknown whether this approach would provide similar outcomes to traditional ADT, and long-term toxicities of [^177^Lu]Lu-PSMA-617, such as increased clonal hematopoiesis mutations, will become more apparent as these treatments are moved to earlier settings (16). Therefore, prospective trials are needed to evaluate the long-term overall survival of these approaches, and the development of new biomarkers that predict response to radiopharmaceutical therapy may allow for more personalized therapy in the future.

## CONCLUSION

A patient with multifocal mHSPC and no prior local or systemic therapy received [^177^Lu]Lu-PSMA-617 as initial therapy. Following 2 cycles, he experienced a 95% reduction in PSA and improvement of urinary symptoms, as well as a durable and near-complete resolution of his prostate cancer primary lesion and nodal and osseous metastases. This treatment strategy adds to the expanding experience utilizing [^177^Lu]Lu-PSMA-617 earlier in the treatment of mHSPC and should prompt additional prospective trials to more fully evaluate its long-term efficacy and safety.

## DISCLOSURE

Marc Garnick is the editor-in-chief of the *Harvard Medical School Guide to Prostate Diseases* and receives an honorarium for that work. He also owns shares in Lantheus, the manufacturer of Pylarify. No other potential conflict of interest relevant to this article was reported.

## Notes

KEY POINTS **QUESTION:** Can metastatic prostate cancer be effectively treated with [^177^Lu]Lu-PSMA-617 before the administration of ADT? **PERTINENT FINDINGS:** We describe the case of a patient with metastatic prostate cancer with many sites of disease who declined ADT, chemotherapy, and primary local treatment to the prostate gland and instead received two cycles of up-front [^177^Lu]Lu-PSMA-617. He continues to experience disease control as of 10 mo from the start of treatment. **IMPLICATIONS FOR PATIENT CARE:** Further investigation is warranted to determine if first-line [^177^Lu]Lu-PSMA-617 can effectively treat metastatic prostate cancer in patients who seek to delay or avoid local treatment to the prostate and systemic therapies, including ADT.
